# The In Vitro Effects of Carprofen on Lipopolysaccharide-Induced Neutrophil Extracellular Trap Formation in Dairy Cows

**DOI:** 10.3390/ani14060985

**Published:** 2024-03-21

**Authors:** Jianbo Zhi, Kaixi Qiao, Lei Xie, Osvaldo Bogado Pascottini, Geert Opsomer, Qiang Dong

**Affiliations:** 1 College of Veterinary Medicine, Northwest A&F University, Yangling, Xianyang 712100, China; zhinwafu@163.com (J.Z.); qkx06100610@126.com (K.Q.); 2 Department of Internal Medicine, Reproduction and Population Medicine, Faculty of Veterinary Medicine, Ghent University, 9820 Merelbeke, Belgium; lei.xie@ugent.be (L.X.); osvaldo.bogado@ugent.be (O.B.P.); geert.opsomer@ugent.be (G.O.)

**Keywords:** neutrophil extracellular traps, carprofen, innate immune function, inflammation, dairy cow

## Abstract

**Simple Summary:**

In the present study, white blood cells (neutrophils) were isolated from the blood of postpartum dairy cows and subjected in vitro to conditions designed to mimic an inflammatory reaction induced by the bacterial toxin named lipopolysaccharide (LPS). The production of neutrophil extracellular traps (NETs), reactive oxygen species (ROS), gene expression of NETs-related proteins and inflammatory factors were quantified. The results indicate that LPS stimulation resulted in a concentration-dependent release of NETs, while ROS exhibited rather minor changes. Nevertheless, the presence of the anti-inflammatory drug Carprofen (CA) resulted in a decrease in NETs formation, a downregulation of gene expression of related proteins as well as pro-inflammatory genes. These results indicate that CA has the potential to serve as a therapeutic for alleviating an excessive inflammatory reaction characterized by an overabundance of NETs. Furthermore, this study illuminates the potential of CA in regulating the immune response and its potential benefits in the treatment of inflammatory conditions in (transition) dairy cows.

**Abstract:**

The objective of this study was to develop an in vitro model that mimics inflammatory reactions and neutrophil extracellular traps (NETs) formation by polymorphonuclear leukocytes (PMNs) in dairy cows. This model was used to examine the effect of carprofen (CA) on lipopolysaccharide (LPS)-induced NETs formation and expression of inflammatory factors. Peripheral blood samples were collected from 24 Holstein cows (3–11 days postpartum) and PMNs were isolated. In three replicates, PMNs were exposed to various treatments to establish an appropriate in vitro model, including 80 μg/mL of LPS for 2 h, followed by co-incubation for 1 h with 60 μmol/L CA and 80 μg/mL LPS. The effects of these treatments were evaluated by assessing NETs formation by extracellular DNA release, gene expression of pro-inflammatory cytokines, reactive oxygen species (ROS) production, and the expression of NETs-related proteins, including histone3 (H3), citrullinated histone (Cit-H3), cathepsin G (CG), and peptidyl arginine deiminase 4 (PAD4). The assessment of these parameters would elucidate the specific mechanism by which CA inhibits the formation of NETs through the PAD4 pathway instead of modulating the Nox2 pathway. This highlights CA’s effect on chromatin decondensation during NETs formation. Statistical analyses were performed utilizing one-way ANOVA with Bonferroni correction. The results demonstrated that LPS led to an elevated formation of NETs, while CA mitigated most of these effects, concurrent the PAD4 protein level increased with LPS stimulating and decreased after CA administration. Nevertheless, the intracellular levels of ROS did not change under the presence of LPS. LPS supplementation resulted in an upregulation of H3 and Cit-H3 protein expression levels. Conversely, the CA administration inhibited their expression. Additionally, there was no change in the expression of CG with either LPS or LPS + CA co-stimulation. The gene expression of pro-inflammatory cytokines (tumor necrosis factor -α, interleukin (IL)-18, IL-1β, and IL-6) upregulated with LPS stimulation, while the treatment with CA inhibited this phenomenon. In conclusion, CA demonstrated a pronounced inhibitory effect on both LPS-induced NETs formation as well as the associated inflammatory response.

## 1. Introduction

Dairy cows experience substantial metabolic and inflammatory alterations in the transition from late gestation to early lactation [1]. These changes contribute to innate immune dysfunction, rendering transition cows vulnerable to a variety of bacterial infections. One of the microbial toxins capable of inducing inflammation is lipopolysaccharides (LPS), which is a constituent of the outer membrane of Gram-negative bacteria and is renowned for its potent inflammatory characteristics. Following exposure to LPS, the immune system of the cow initiates a multifaceted cascade of inflammatory reactions [2].

One of the remarkable functionalities of polymorphonuclear leukocytes (PMNs) is to capture and neutralize pathogens by releasing neutrophil extracellular traps (NETs), often referred to as “NETosis” [3]. NETs encompass intricate webs comprising DNA and antimicrobial proteins that can efficiently immobilize and eliminate bacteria [4]. During infection with Gram-positive bacteria such as *Staphylococcus aureus*, phagocytosis of PMNs initiates bacterial killing through the production of reactive oxygen species (ROS) by NADPH (nicotinamide adenine dinucleotide phosphate) oxidase [5,6] or fusing granules containing antimicrobial peptides to the phagosome [7,8]. Nevertheless, dysregulated formation of NETs can result in tissue injury and chronic inflammation [9]. 

Previous studies have shown that LPS can stimulate the release of NETs in a concentration-dependent manner [10,11,12]. However, the LPS concentration and duration of exposure showed variability across different in vitro studies [13,14,15,16]. These differences may be attributed to inherent variations in the enzymatic function of PMNs granules among distinct species [17]. Remarkably, the process of NETs formation coincides with the generation of ROS, therefore, the measurement of ROS levels may serve as an indirect approach to evaluate NETs production in vitro [10,14]. For research purposes, the quantification of NETs-associated proteins, including citrullinated histone H3 (Cit-H3), peptidyl arginine deiminase 4 (PAD4), histone3 (H3), and cathepsin G (CG) can be used as NETs-generated indicators [18,19]. Furthermore, the assessment of pro-inflammatory cytokines such as tumor necrosis factor (TNF)-α, interleukin (IL)-6, IL-18, and IL-1β by PMNs can offer valuable insights into the potential therapeutic impact of non-steroidal anti-inflammatory drugs (NSAIDs) on the reduction of excessive inflammation associated with persistent NETs formation in transition cows [20,21].

Carprofen (CA), as one of NSAIDs, has garnered extensive usage in dairy cattle to alleviate inflammation and mitigate pain [22,23]. Its therapeutic outcome predominantly stems from its capacity to impede the enzymatic activity of cyclooxygenase (COX)-2, thereby diminishing the synthesis of pro-inflammatory mediators such as prostaglandins [24]. Ketoprofen, as an NSAID, can inhibit TNF-α, IL-1β, and IL-6 in LPS-stimulated porcine PMNs [25]. Meanwhile, meloxicam attenuates LPS-induced TNF-α in adult donkey monocytes and bovine mammary cells [26,27]. However, the precise impact of CA on the formation of NETs continues to be a subject of investigation, underscoring its involvement in crucial immune response mechanisms relative to inflammation.

Up until today, whether CA can modulate the formation of NETs and have an effect on LPS-induced inflammatory responses of PMNs is unclear. Therefore, the in vitro experiments described in the present study aimed to achieve two main objectives, (1) to determine the optimal concentrations and incubation time of LPS and CA to establish an LPS model, focusing on PMN viability, ROS production, and NETs formation, and to (2) utilize the established LPS model to evaluate the impact of CA on LPS-induced NETs formation, as well as the expression of NETs-related proteins and inflammatory cytokines in PMNs. In practical terms, our objective was to construct a model that explores the potential use of CA as an immune modulator in managing infectious, dysfunctional inflammation during the transition period of dairy cows.

## 2. Materials and Methods

### 2.1. Ethical Statement

All animal handling and sampling procedures were approved by the Animal Care Commission of the College of Veterinary Medicine, Northwest A&F University, China Ethical approval for this study was obtained from the Animal Care Commission under protocol number NWLA-DY2022-048. The approval for the study was granted in March 2022.

### 2.2. Animals and Management

In the present experiment, cows were provided by Aohua Modern Animal Husbandry (E 107°55′31.9476″, N 34°11′30.8868″), Meixian, China. From May to December 2022, a total of 24 Holstein cows within 3–11 days in milk (DIM) were selected based on the following inclusion criteria: serum non-esterified fatty acid (NEFA) concentration ranging from 0.6 to 1.0 mmol/L, serum β-hydroxybutyrate (BHB) levels ≤ 0.9 mmol/L, and blood glucose levels ranging from 2.0 to 3.8 mmol/L. All the cows were clinically healthy, without fever, postpartum metritis, ketosis, or other clinical diseases. In the early post-calving, the average daily milk yield of the sampled cows was 31.0 kg (29.37–48.5 kg), the Dry Matter Intake was 16.4 kg, the energy density of the daily rations provided was 1.56 Mcal/kg, and the ratio of forage to concentrates was 53.7:46.3. After calving, cows were transferred to a free-stall lactating pen and milked three times a day. Cows were provided with a total mixed ration three times a day at 7:00 a.m., 1:30 p.m., and 7:30 p.m. All cows were given unrestricted access to water.

### 2.3. Reagents and Materials

Trypan blue, Triton X-100, 2,7-dichlorofluorescein diacetate (DCFH-DA), lipopolysaccharide from *E. coli O55:B5* (LPS) (L2880), Carprofen (PHR1452) and 3, 3′, 5, 5′-Tetramethylbenzidine (TMB) (860336) were purchased from Sigma-Aldrich (Shanghai, China). SYTOX Orange dye (S11368), Quant-IT™ Pico-Green dsDNA Assay kit (P7589) and transparent 24-well plates were obtained from Thermo-Fisher Scientific Technology Co., Ltd. (Shanghai, China). Roswell Park Memorial Institute (RPMI) 1640 dry powder and phenol red-free RPMI 1640 medium were purchased from Thermo-Fisher Scientific Technology Co., Ltd (Shanghai, China). Polylysine, Giemsa stain kits and LDH Cytotoxicity Assay Kit were purchased from Jiangsu Beyotime Biotechnology Co., Ltd. (Nanjing, China). SuperReal PreMix plus (SYBR Greenand FastKing RT kit were obtained from TianGen Biotechnology Co., Ltd. (Beijing, China). RNAiso Plus was acquired from TaKaRa Bio Co., Ltd. (Shiga, Japan) Holoprotein extraction kit, BCA Protein Assay kit, Tris-HCl, and PAGE Pre-Solution were purchased from Solarbio Life Sciences (Beijing, China). Black 96-well plates were purchased from Shanghai Jing’an Biological Technology Co., Ltd. (Shanghai, China).

### 2.4. Experiment 1: Effects of CA and LPS on Circulating PMNs Viability, PMNs Production of NETs and ROS

**Figure 1:**
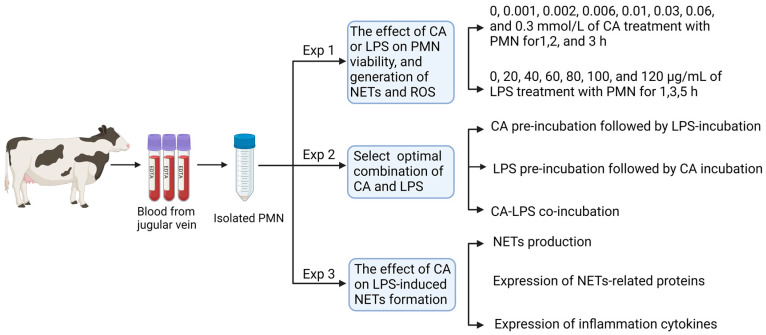
Schematic overview of the experimental design. Polymorphonuclear neutrophils (PMNs) were isolated from blood samples collected in EDTA tubes, with each of the three replicates undergoing three separate experiments. Experiment 1 focused on studying the effects of carprofen (CA) and lipopolysaccharide (LPS) supplementation on PMN viability, as well as the production of neutrophil extracellular traps (NETs) and reactive oxygen species (ROS). Experiment 2 involved selecting the optimal combinations of CA and LPS based on PMN viability. Experiment 3 examined the impact of CA on the formation of LPS-induced NETs, NETs-related proteins, and inflammatory cytokines.

This section aimed to select the optimal concentration and culture duration of CA and LPS in PMNs suspension. To do so, blood samples were collected from three randomly selected cows and PMNs were isolated. Samples were processed in triplicate. The study design of this section is shown in Figure 1.

#### 2.4.1. Blood Sample Collection and PMNs Isolation

Blood samples were collected from the jugular vein of each cow using BD Vacutainer Plus tubes without anticoagulant (n = 2) and EDTA tubes (n = 5) (Franklin Lakes, NJ, USA) at 6:30 a.m. After collection, the blood samples were promptly transported to the laboratory within 1 h and subsequently centrifuged at 600× *g* for 15 min at 4 °C. Then, the collected serum was stored at −80 °C for subsequent assessment of biochemical metabolic parameters. The EDTA tubes were used for PMNs isolation. PMNs isolation was conducted following the method outlined by Xie et al. [10]. Cell viability was assessed using Trypan blue solution, and Giemsa staining was employed to evaluate cell purity [28].

To determine the optimal dose and incubation duration of CA, PMNs were treated with 0, 0.001, 0.002, 0.006, 0.01, 0.03, 0.06, and 0.3 mmol/L CA for 1, 2, and 3 h. For LPS, the PMNs were stimulated with 0, 20, 40, 60, 80, 100, and 120 μg/mL LPS for 1, 3, and 5 h. In brief, 100 μL RPMI 1640 medium containing 6 × 10^5^ PMNs were seeded in triplicate in a 96-well plate and incubated at 37 °C with 5% CO_2_ for 30 min. Then incubated with 100 μL of the specific concentration of CA or LPS at specific intervals as outlined above.

#### 2.4.2. Determination of PMNs Viability

Neutrophil viability was assessed using a lactate dehydrogenase (LDH) cytotoxicity detection kit complying with the manufacturer’s instructions. Briefly, 100 μL culture medium containing 6 × 10^5^ PMNs/well were seeded into a 96-well plate, incubated at 37 °C with 5% CO_2_ for 30 min, and then exposed to CA or LPS at concentrations and for durations as outlined in Section 2.4.1. One hour before the detection, the LDH release reagent was added, gently mixed, and moved back to incubation for 1 h. After incubation, the plate was centrifuged at 400× *g* for 5 min, and 120 μL of the supernatant was transferred to a new 96-well plate, followed by adding 60 μL of LDH detection working solution and incubated at room temperature for 30 min in the dark. Ultimately, the Microplate Spectrophotometer (BioTek Epoch, Winooski, VT, USA) was used to measure absorbance at 490 nm. The viability was calculated by the formula: cell viability (% viable cells) = 100 − (absorbance of treated sample − absorbance of sample control well)/(absorbance of maximum cell enzyme activity − absorbance of sample control well) × 100%. The optimal concentration was defined as the highest possible concentration of CA or LPS safeguarding the highest viability of the PMNs.

#### 2.4.3. Detection of ROS Production

The detection of ROS production was performed according to the method outlined by Xie et al. [10]. Briefly, PMNs were diluted with RPMI 1640 medium, and 200 µL containing 6 × 10^5^ cells was subsequently inoculated into 96-well plates and cultured with 0, 20, 40, 60, 80 μg/mL LPS for 3 h at 37 °C and 5% CO_2_.

#### 2.4.4. Quantification of NETs

The Quant-iT™ PicoGreen dsDNA Assay kit was employed to quantify the NETs level according to the method outlined by Xie et al. [10]. Briefly, PMNs were diluted with RPMI 1640 medium, and 200 µL containing 6 × 10^5^ cells was subsequently inoculated into 96-well plates and cultured with 0, 20, 40, 60, and 80 μg/mL LPS for 1 and 3 h at 37 °C and 5% CO_2_.

### 2.5. Experiment 2: Selecting the Optimal Combination of CA and LPS Incubation Based on PMNs Viability

**Figure 2:**
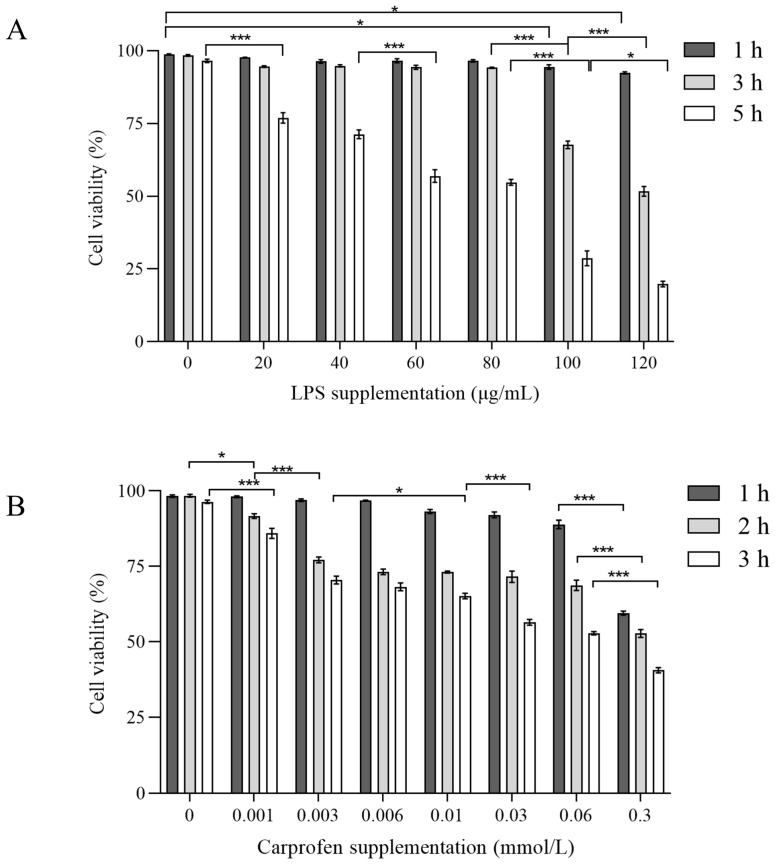
The effect of lipopolysaccharide (LPS) and Carprofen (CA) on the neutrophil viability. Neutrophil viability was assessed by measuring LDH enzyme activity. (**A**) Neutrophils were treated with LPS (0, 20, 40, 60, 80, 100 and 120 µg/mL) for 1, 3, and 5 h. (**B**) Neutrophils were treated with CA (0, 0.001, 0.003, 0.006, 0.01, 0.03, 0.06 and 0.3 mmol/L) for 1, 2 and 3 h. Experiments were performed three times and in triplicate per time. Each column represents the mean ± SEM. Statistical significance is indicated as follows: *** represents *p* < 0.001, * represents *p* < 0.05.

**Figure 3:**
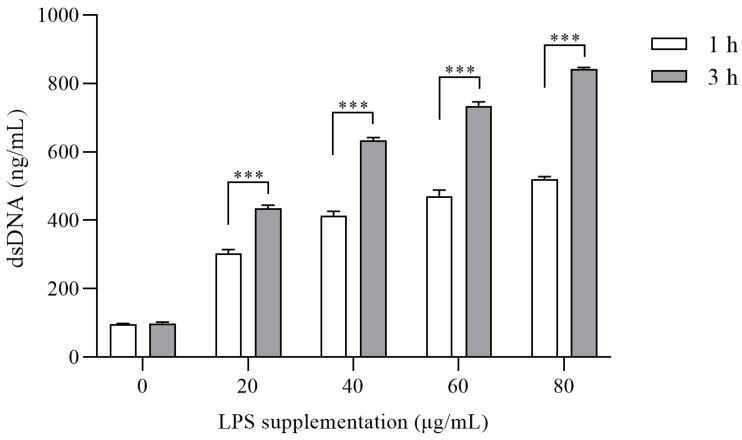
The effect of various concentrations of lipopolysaccharide (LPS) for 1 and 3 h on neutrophil extracellular traps (NETs; dsDNA) level. Neutrophils were treated with 0, 20, 40, 60, and 80 μg/mL LPS for 1 and 3 h. dsDNA was quantified by the Quant-iT™ PicoGreen dsDNA assay kit. Each column represents the mean ± SEM. *** represents *p* < 0.001.

Based on the results of Experiment 1, the optimal concentrations and culture times for CA (60 μM, 1 h) and LPS (80 μg/mL, 1 h and 3 h) were selected (Figure 1, Figure 2 and Figure 3). In this section, we aimed to select the optimal combination of CA and LPS supplemented with PMNs suspensions, searching for the combination with lesser effects on PMNs viability. To do so, blood samples were collected from three randomly selected cows and PMNs were isolated. Samples were processed in triplicate. The design of this section is shown in Figure 1.

Three treatment groups were designed to identify the most optimal way to combine the CA and LPS in PMNs suspensions: CA pre-incubation followed by LPS-incubation, LPS pre-incubation followed by CA incubation, or CA-LPS co-incubation. For all groups, 200 μL comprising 6 × 10^5^ cells were incubated in 96-well plates at 37 °C and 5% CO_2_ with different combinations of CA and LPS. For the CA pre-incubation (CP) group, PMNs suspension with 60 μM of CA was added to each well. After 1 h of incubation and then centrifugation at 500× *g* for 4 min at 25 °C, the culture medium was discarded, and the cells were washed twice using 1 x PBS. After that, 200 μL (80 μg/mL) LPS solution was added for an extra 3 h incubation. For the LPS pre-incubation (LP) group, 80 μg/mL of LPS was added to a PMNs suspension in each well for 1 h incubation. After centrifugation at 500× *g* for 4 min at 25 °C, the culture medium was discarded, and cells were washed twice. Then, 200 μL (60 μM) CA was added for 1 h incubation. For the LPS and CA co-incubation (LC-Co) group, 80 μg/mL of LPS was added to the PMNs suspension in each well and incubated for 2 h. After centrifugation at 500× *g* for 4 min at 25 °C, the culture medium was discarded, and cells were washed twice using 1 x PBS and a final 200 μL 1640 medium containing 80 μg/mL LPS and 60 μM CA was added for an extra 1 h co-incubation. The LDH cytotoxicity detection kit was used to assess the viability of PMNs (see Section 2.4.2). The incubation combination with the lowest effect on the PMNs viability was finally selected for detecting proteins and inflammation cytokines.

### 2.6. Experiment 3: The Effects of LPS and CA Co-Incubation (LC-Co) on PMNs’ NETs Formation, Protein and Inflammatory Factors’ Expression

Based on the results achieved in Experiment 2, the optimal combination of CA and LPS treatment was found to be their co-incubation (LC-Co group). We aimed to investigate the effects of LC-Co on PMNs’ NETs formation, NETs-related protein, and the gene expression of inflammatory factors. To do so, blood samples from three randomly selected cows were used, PMNs were isolated, and proteins and RNA were extracted and quantified. Samples were processed in triplicate. To determine the impact of the LC-Co induced NETs production, various analytical approaches were used. The level of NETs was quantified using the Quant-IT™ Pico-Green assay (see Section 2.4.4). The morphological visualization of NETs was performed using the SYTOX Orange fluorescent staining followed by ImageJ (V1.8.0.112) for visual assessment. Protein expression was assessed via western blotting. Inflammatory related-gene expressions were measured using the real-time fluorescence quantitative technique. The study design of this section is shown in Figure 1.

For the assessment of the microscopic visualization of NETs, two groups sets were created, one with PMNs treated with 80 μg/mL LPS followed by incubation at 37 °C with 5% CO_2_ for 3 h. The second group was similar to the LC-Co group in Experiment 2: 80 μg/mL of LPS was added to the PMNs suspension in each well and incubated for 2 h. Subsequently, the culture medium was discarded, 80 μg/mL of LPS and 60 μmol/L CA was added and incubated for 1 h. For the analysis of NETs-related proteins and inflammatory factors, 6-well plates were used, with 2 mL of RPMI 1640 containing 1 × 10^6^ cells per well.

#### 2.6.1. Morphological Observation and Quantification of NETs

Polylysine-coated cell culture climbing sheets were placed in 24-well plates in advance. Then, 1 mL of RPMI medium containing 5 × 10^4^ PMNs was seeded. The plates, covered with lids, were cultured at 37 °C in 5% CO_2_ for 3 h to stimulate cell adhesion to the poly-lysine cell climbing sheets. After incubation, the cells were fixed with 4% paraformaldehyde for 30 min and then washed three times for 3 min with PBS. Subsequently, 500 μL of 0.3% Triton X-100 (diluted in 1 x PBS) was added to facilitate permeabilization for 15 min at room temperature, followed by washing with PBS. Then 500 μL of 5 μM Sytox Orange, dissolved in PBS, was added to each well and incubated for 8 min in the dark. After incubation, the residual dye was discarded, and the slides were removed and washed with PBS. Finally, the sheets were examined and photographed using a fluorescence inverted microscope (Carl Zeiss, Ober-kochen, Germany). ImageJ software (https://imagej.net/ij/) was used to analyze the average fluorescence intensity of 8 randomly selected images from each sample.

#### 2.6.2. Protein Extraction and Western Blotting

After incubation, total protein extraction was carried out using protein extraction kits and protein concentrations were determined using protein assay kits. Proteins were transferred to PVDF membranes (Millipore, Burlington, MA, USA) and blocked by TBST (50 mmol/L Tris-HCl, pH 8.0, 5 mol/L NaCl, and 0.1% Tween 20) containing 5% non-fat milk powder for 2 h at room temperature. The immunoblotting was performed with primary antibodies, including H3, Cit-H3 (citrulline R2 + R8 + R17), CG, and PAD4 (stored at 4 °C overnight). Then, the membrane was washed three times using TBST, and the horseradish peroxidase (HRP)-conjugated secondary antibody (diluted in TBST) was added and incubated for 2 h at room temperature. To visualize the protein bands, the membrane was exposed to the Amersham ImageQuant 800 biomolecular imager (Cytiva, Tokyo, Japan) using enhanced chemiluminescence (DiNING, Beijing, China). The relative intensity of each band was assessed using Image J software. Table 1a,b show the primary antibodies and HRP-conjugated secondary antibodies used in the present study. 

#### 2.6.3. RNA Extraction and Quantitative Real-Time PCR

After incubation, total RNA was extracted using the RNAiso Plus kit, and the RNA concentration was measured using a NanoDrop ND-1000 Spectrophotometer (NanoDrop, Wilmington, NC, USA). One μg of RNA was added for the genomic DNA elimination reaction and reverse transcription into a cDNA template using the FastKing RT Kit. The primer sequences were synthesized by Tsingke Biotechnology Co., Ltd. (Beijing, China) (Table 2). Quantitative PCR was conducted using the CFX-connect system and the SYBR Green Plus Reagent Kit. The reaction conditions complied with 95 °C for 15 min, 40 cycles at 95 °C for 10 s and 60 °C for 27 s. The relative expression of genes was calculated using the 2^−ΔΔCT^ method. β-actin was used as a reference gene to standardize the expression levels of the other genes.

### 2.7. Statistical Analysis

All data are reported as the mean ± standard deviation (SD). Statistical analyses were conducted using SPSS 20 (International Business Machines Corporation, Armonk, NY, USA). One-way analysis of variance (ANOVA) was used to compare multiple groups. Figures were created using the GraphPad Prism 9.0 software (GraphPad Software Inc., La Jolla, CA, USA). Fluorescence images were processed with ZEN Microscopy Software v.2.3 (Zeiss, Oberkochen, Germany), and the images were further processed using Image J software (National Institutes of Health, Bethesda, MD, USA). Significant differences: ns represents *p* ≥ 0.05, * indicates *p* < 0.05, ** indicates *p* < 0.01, *** indicates *p* < 0.001.

## 3. Results

### 3.1. Experiment 1: Effects of CA and LPS on PMNs Viability, PMNs Production of ROS and NETs

The results of the PMNs’ viability treated with different doses of LPS or CA are shown in Figure 2A,B. There was no change in viability when PMNs were exposed to 0–80 μg/mL LPS for 1 h (*p* > 0.05). Viability decreased when LPS concentrations reached 100 and 120 μg/mL (*p* = 0.01 and *p* = 0.03 respectively). After 3 h incubation, the PMNs viability decreased when LPS concentration reached 100 μg/mL in comparison to 80 μg/mL (*p* < 0.0001). For CA treatment, a concentration > 0.06 mmol/L decreased PMNs viability after 1 h incubation (*p* < 0.0001). As the exposure duration to 0.001 mmol/L CA increased to 2 or 3 h, there was a notable reduction in viability compared to the non-CA group (*p* = 0.01 and *p* < 0.0001 respectively). PMNs maintained good viability under conditions of LPS exposure up to 80 μg/mL at 1 and 3 h, or with CA treatment at 0.06 μmol/L for 1 h respectively.

The supplementation of 20, 40, 60, and 80 μg/mL of LPS resulted in minor changes in ROS production when compared to the non-LPS group after 3 h of treatment (*p* > 0.2). Results about the quantitative assessment of double-stranded DNA produced by NETs are shown in Figure 3. The quantification of dsDNA was determined using the standard formula: dsDNA = (NETs Fluorescence value − 84.52)/50.679. The stimulation of PMNs with LPS dose-dependently increased following 1 or 3 h of incubation. LPS have little impact following a 1 h incubation but a significant effect on dsDNA level after 3 h (*p* < 0.0001).

### 3.2. Experiment 2: The Optimal Combination of CA and LPS Incubation Based on PMNs Viability 

The PMN viability was determined after their supplementation with LPS and CA at various concentrations. Compared to the LPS group (87.2 ± 2.3%), neutrophil viability decreased in the first CA-pre-incubation group (51.5 ± 4.0) (*p* < 0.0001) and first LPS-pre-incubation group (57.7 ± 2.5%) (*p* < 0.0001). The PMNs viability had a minor change when treated with LPS-CA co-incubation (81.9 ± 0.7%) in comparison to LPS group (*p* = 0.19).

### 3.3. Experiment 3: The Effects of LPS and CA Co-Incubation (LC-Co) on PMNs’ NETs Formation, Protein and Inflammatory Expression

**Figure 4:**
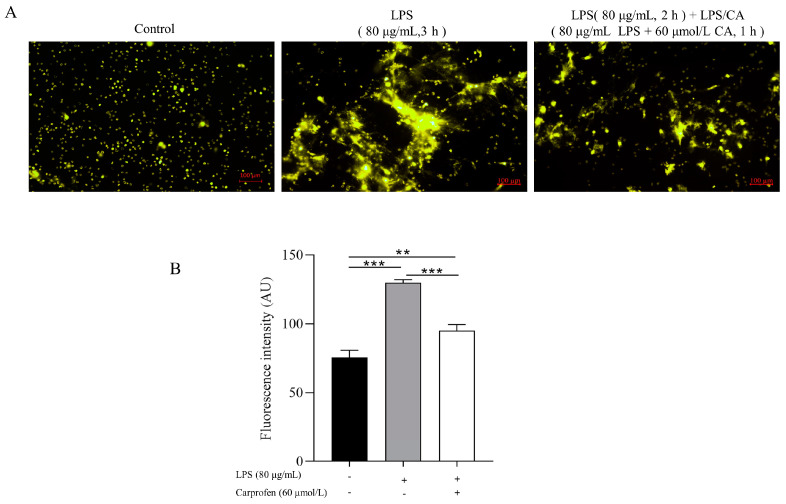
Representative fluorescence microscopy images of neutrophil extracellular traps (NETs) stained with SYTOX orange of neutrophils treated with lipopolysaccharide (LPS) and LPS + Carprofen (CA) for 3 h. Neutrophils were treated with LPS, and LPS + CA co-incubation for 3 h as described in the figure. The quantification of eight randomly selected images from each group was performed using ImageJ software. Figure (**B**) represents the results quantified from Figure (**A**). Each column represents the mean ± SEM. *** represents *p* < 0.001, ** represents *p* < 0.01.

Representative images and quantification of NETs formation treated with LPS and LPS-CA co-incubation are shown in Figure 4A,B. Figure 4B represents the results quantified from Figure 4A, indicating NETs formation in the LPS + CA co-incubation group (95.0 ± 4.5 AU) decreased compared to the LPS group (129.9 ± 2.1 AU) (*p* < 0.0001). 

**Figure 5:**
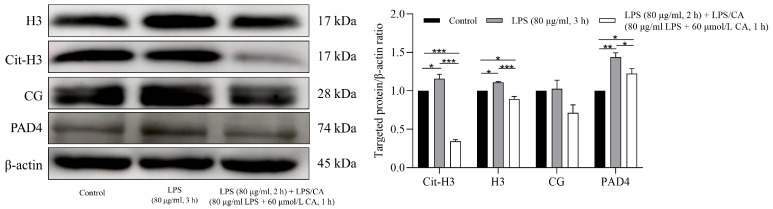
The effects of lipopolysaccharides (LPS) and LPS + Carprofen (CA) co-incubation for 3 h on neutrophil extracellular traps (NETs)-related protein expression. The expressions of proteins were assessed through western blotting and the results were quantified by ImageJ software. All data originate from three biological replicates. Each column represents the mean ± SEM.*** represents *p* < 0.001, ** represents *p* < 0.01, * represents *p* < 0.05.

The results of the western blotting analysis of NETs-related protein expression treated with LPS and LPS-CA (LC-Co) co-incubation for 3 h are shown in Figure 5. Compared to the non-LPS group, the LPS group showed increased expressions of H3, Cit-H3 and PAD4 (*p* = 0.02, *p* = 0.04 and *p* = 0.001 respectively), with no increased expression of CG (*p* = 0.9). In the LC-Co group, the expressions of Cit-H3, H3, and PAD4 were decreased (*p* < 0.0001, *p* = 0.0009 and *p* = 0.04 respectively) compared to the LPS group, while the expression of CG tended to be decreased (*p* = 0.09). 

**Figure 6:**
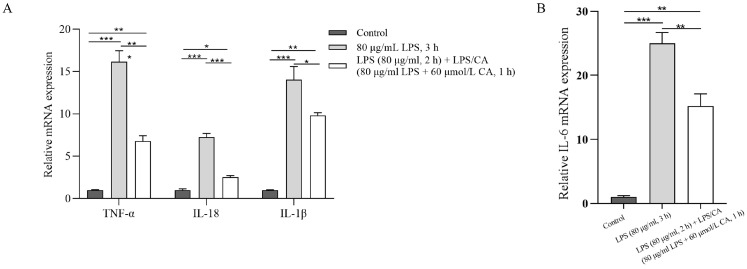
The effect of lipopolysaccharide (LPS) and LPS + Carprofen (CA) co-incubation for 3 h on the expression of pro-inflammatory cytokines (Tumor necrosis factor-α (TNF-α), interleukin-6 (IL-6), IL-18 and IL-1β) by neutrophils. Gene expression was assessed through the Real-time qPCR technique and all gene expressions were normalized to β-actin. All data originate from three biological replications. Figure (**A**) shows the mRNA expression levels of TNF-a, IL-18 and IL-1B, and Figure (**B**) shows the mRNA expression level of IL-6. Each column represents the mean ± SEM. *** represents *p* < 0.001, ** represents *p* < 0.01, * represents *p* < 0.05.

Figure 6 shows the mRNA expression of inflammatory cytokines in PMNs incubated with LPS and LPS-CA. The gene expression of TNF-α, IL-18, IL-1β, and IL-6 upregulated in the LPS group compared to the non-LPS group (*p* < 0.0001, *p* < 0.0001, *p* = 0.0001, *p* < 0.0001, respectively). However, the addition of CA downregulated the LPS-induced gene expressions (*p* = 0.0005, *p* < 0.0001, *p* = 0.0421, *p* = 0.0079, respectively).

## 4. Discussion

In the present study, we opted for an LPS concentration of 80 μg/mL and a duration of incubation of 3 h to establish an inflammatory and NETs formation model by circulating PMNs isolated from postpartum dairy cows. The model demonstrates that PMNs respond to LPS stimulation by releasing pro-inflammatory factors like TNF-α, IL-6, IL-18, and IL-1β and forming NETs through the PAD4 pathway. After introducing CA to this LPS model, a decreased NETs formation and downregulated expression of inflammatory factors was observed, along with proteins related to the PAD4 pathway.

In vitro models of inflammation have been effectively developed using 100 μg/mL LPS for 1 h or 80 μg/mL LPS for 3 h [10,21]. To ensure the reliability of our model, cell viability measurements were employed to eliminate the interference of deceased cells. In our inflammation model, PMNs showed a significant upregulation of TNF-α, IL-6, IL-18, and IL-1β expression, which is consistent with previous outcomes [10,21]. The results collectively demonstrate the reproducibility of LPS stimulation in the in vitro bovine PMNs inflammation model. Even within the same species, the NETs formation ability of PMNs at different physiological stages can vary [10]. The present study also revealed that a simultaneous incubation of LPS-CA with PMNs had the best effect compared to CA pre- and post-incubation relative to the LPS challenge. 

Aligned with the increase of pro-inflammatory factors, LPS triggered the formation of NETs, and CA attenuated this effect. Previous studies have shown that LPS can activate NF-κB and MAPK through the TLR/MyD88 pathway, resulting in the production of pro-inflammatory cytokines [20,21]. Furthermore, it has been observed that NETs induced by LPS may impact the TLR4/NF-κB pathway [29]. Hence, a preliminary conclusion can be drawn that CA has the potential to alleviate the inflammatory response and concomitant NETs formation triggered by LPS.

Two primary pathways are recognized for the formation of NETs [30,31]. One is the activation of NADPH oxidase 2 (Nox2), resulting in the production of ROS (Nox2-dependent pathway) [31]. Another mechanism involves the activation of PAD4 via increased Ca^2+^ levels, which leads to histone decondensation [32]. This PAD4 pathway occurs independently of the Nox2 pathway [33,34,35]. The present study demonstrates increased PAD4 and citrullinated histone H3 (Cit-H3) levels in LPS-activated PMNs, with no change in ROS production, suggesting NETs generation by LPS may not depend on the Nox2 pathway, instead on the Ca^2+^-PAD4 pathway. Moreover, we observed upregulation of LPS-induced Cit-H3 and histone H3 and their downregulation following CA treatment, but no change in PAD4 expression. Multiple studies have demonstrated PAD4 to be involved in mediating histone citrullination [14,36]. We can presume that CA may potentially reduce NETs by inhibiting H3 expression, but we cannot completely dismiss the regulatory role of PAD4 in the histone citrullination process. Additionally, there was no change in the expression of Cathepsin G (CG) protein with either LPS or CA supplementation. It has been reported that the primary proteolytic activity involved in NETs formation is attributed to NE. When NE was immunodepleted, the residual activity was attributed to CG [37]. The latter indicates that CG protein may not play a primary regulatory role in the process of NETs formation. Therefore, it is possible that CA can effectively modulate NETs formation via the PAD4 pathway. Understanding how CA diminishes the formation of NETs and inflammation, holds direct implications for managing postpartum health and preventing inflammatory complications in transition dairy cows.

The concurrent supplementation of LPS and CA suggests a potential therapeutic role for CA in mitigating infectious, dysfunctional inflammation. These findings prompt further exploration into the dose-dependent effects of CA on NETs formation and overall immune function. Additionally, it highlights the need for strategies to attenuate excessive NETs formation in vivo and the potential utilization of CA as a therapeutic agent to control the harmful (side) effects of chronic NETosis.

## 5. Conclusions

In conclusion, this study has effectively established an in vitro model for inducing stable PMN inflammation in dairy cows and suggests that LPS-induced NETs formation occurs via the PAD4 signaling pathway. Additionally, CA was found to inhibit NETs formation and concomitant pro-inflammatory cytokines, which is crucial in preventing systemic inflammation. Further exploration is necessary to determine regulatory mechanisms and assess the potential clinical uses of CA in preventing infectious inflammation in dairy cows.

## Figures and Tables

**Table 1:** (a) Primary antibodies used in the western blotting experiment. (b) Horseradish peroxidase-conjugated secondary antibodies used in the western blotting experiment.

**a**			
**Antigen**	**Host**	**Manufacturer**	**Dilution**
Anti-PAD4	Rabbit	Abcam (#ab214810)	1:5000 (WB)
Anti-Cathepsin G	Rabbit	Abcam (#ab282105)	1:5000 (WB)
Anti-Histone H3	Rabbit	Abcam (#ab32356)	1:5000 (WB)
Anti-Histone H3 (citrulline R2 + R8 + R17)	Rabbit	Abcam (#ab5103)	1:5000 (WB)
β-actin	Mouse	Sungene Biotech (KM9001T)	1:5000 (WB)
**b**			
**Antigen**	**Host**	**Manufacturer**	**Dilution**
HRP-Conjugated Goat Anti-Rabbit	Rabbit	Beyotime (A0208)	1:2000 (WB)
HRP-Conjugated Goat Anti-Mouse	Mouse	Beyotime (A0216)	1:2000 (WB)

**Table 2:** Primer sequences for qRT-PCR.

Genes	Accession No.	Accession No.	Products (bp)
*β-actin*	NM_173979.3	F: TGAGGCTCAGAGCAAGAGAGG R: AGGCATACAGGGACAGCACA	266
*TNF-α*	NM_173966.3	F: CTCCTTCCTCCTGGTTGCAG R: CACCTGGGGACTGCTCTTC	92
*IL-6*	NM_173923.2	F: AGCGCATGGTCGACAAAATC R: CCCAGATTGGAAGCATCCGT	143
*IL-18*	NM_174091.2	F: GATTATTGCATCAGCTTTGTGGAAA R: GATCTGATTCCAGGTCTTCATCAT	88
*IL-1β*	NM_174093.1	F: AAAATCCCTGGTGCTGGCTA R: AGCTCATGCAGAACACCACT	92

## Data Availability

The data that support the findings of this study is available on the request from the corresponding author.
